# Bibliographical

**Published:** 1880-06

**Authors:** 


### BIBLIOGRAPHICAL.

The Black Arts in Medicine, with anniversary address, by John D. Jackson, A. M., M. D. Edited by L. S. McMurtry, A. M., M. D.

This work consists mainly of a sharp criticism upon some of the modes and practices of professional men, especially physicians, employed in procuring and retaining business. The subject is presented in a letter by Dr. Solomon Machiavelli Sharp, A. B., A. M., M. D., etc., etc., to John Charlatan Greene, M. D. The points discussed are such as educated and observing persons are somewhat familiar with, yet, judging by the success attained by some who adopt questionable methods, there are many in every community who either enjoy being humbugged, or are very deficient in observation and sagacity in such matters. The criticisms will apply equally as well to dentists as physicians, for many of the "black arts" employed by the latter are also used by the former, and are alike reprehensible in both.

The book ought to be read by everybody, and especially by the people. It is for sale by Robt. Clark & Co., of this city.

